# Extraction of Chitin, Chitosan, and Calcium Acetate from Mussel Shells for Sustainable Waste Management

**DOI:** 10.3390/ijms26157107

**Published:** 2025-07-23

**Authors:** Chaowared Seangarun, Somkiat Seesanong, Banjong Boonchom, Nongnuch Laohavisuti, Pesak Rungrojchaipon, Wimonmat Boonmee, Sirichet Punthipayanon, Montree Thongkam

**Affiliations:** 1 Material Science for Environmental Sustainability Research Unit, School of Science, King Mongkut’s Institute of Technology Ladkrabang, Bangkok 10520, Thailand; chaowared@gmail.com; 2 Office of Administrative Interdisciplinary Program on Agricultural Technology, School of Agricultural Technology, King Mongkut’s Institute of Technology Ladkrabang, Bangkok 10520, Thailand; somkiat.se@kmitl.ac.th; 3 Municipal Waste and Wastewater Management Learning Center, School of Science, King Mongkut’s Institute of Technology Ladkrabang, Bangkok 10520, Thailand; 4 Department of Chemistry, School of Science, King Mongkut’s Institute of Technology Ladkrabang, Bangkok 10520, Thailand; pesak.ru@kmitl.ac.th (P.R.); montee.th@kmitl.ac.th (M.T.); 5 Department of Animal Production Technology and Fishery, School of Agricultural Technology, King Mongkut’s Institute of Technology Ladkrabang, Bangkok 10520, Thailand; nongnuch.la@kmitl.ac.th; 6 Department of Biology, School of Science, King Mongkut’s Institute of Technology Ladkrabang, Bangkok 10520, Thailand; wimonmat.bo@kmitl.ac.th; 7 Department of Sports Science, Faculty of Physical Education, Srinakharinwirot University, Bangkok 10110, Thailand

**Keywords:** chitin, chitosan, calcium acetate, mussel shells, waste management

## Abstract

In this paper, mussel shells were used to produce chitin, chitosan, and calcium acetate using chemical processes, searching for an alternative environmentally friendly biopolymer and calcium source. Mussel shells were treated with acetic acid as a demineralizing agent, resulting in separate solid fractions and calcium solution. The solid was further purified to produce chitin by deproteinization and decolorization processes, and then the deacetylation process was used to obtain chitosan. The calcium solution was evaporated to produce calcium acetate powder. The yields of extracted chitin, chitosan, and calcium acetate from 100 g of mussel shells were 2.98, 2.70, and 165.23 g, respectively. The prepared chitin, chitosan, and calcium acetate were analyzed by Fourier transform infrared (FTIR) spectrophotometry, X-ray diffraction (XRD), thermogravimetric analysis (TGA), and scanning electron microscope (SEM) to confirm the chemical and physical properties. The analysis results of chitin and chitosan revealed the similarity to chitosan derived from crustaceans and insects in terms of functional group, structure and morphologies. The prepared calcium acetate shows FTIR and XRD data corresponding to calcium acetate monohydrate (Ca(CH_3_COO)_2_·H_2_O) similar to synthesized calcium acetate in previous research. In addition, the mineral contents of calcium acetate identified by X-ray fluorescence (XRF) analysis exhibit 97.8% CaO with non-toxic impurities. This work demonstrated the potential of the production process of chitin, chitosan, and calcium acetate for the development of a sustainable industrial process with competitive functional performance against the commercial chitin and chitosan production process using crustacean shells and supported the implementation of a circular economy.

## 1. Introduction

Mussels, an economic seafood product, have been produced worldwide, amounting to more than 2 million tons per year [1]. After the food production processes, around 70% of the total mussel weight was discarded as mussel shell waste in large amounts [2]. As a result, discarded mussel shells are dumped into landfills or in the sea without control of the discarded shells, damaging the ecosystems of soil, water, and air [3] and leading to various environmental and health problems [4]. Alternatively, waste materials offer significant advantages when used as renewable resources for producing value-added materials, leading to various benefits in both economic and environmental aspects [5]. It has been reported that mussel shells mainly contain more than 94% of calcium carbonate (CaCO_3_) [6,7] and 6% of others as organic matrices, including chitin [8,9], pointing to alternative sources of biopolymer and calcium to produce chitin/chitosan and advanced calcium compounds. Chitin, a natural polysaccharide made from *N*-acetyl-D-glucosamine units, is the second most abundant biopolymer on Earth after cellulose. Chitin has three allomorphs, namely the α, β, and γ forms, depending on the source of chitin [10]. In nature, chitin can be found in crustacean shells, mussel shells, insects, and microorganisms such as fungi, algae, and yeasts [2,11]. Due to its strong characteristic structure and favorable properties, chitin was considered a possible replacement for petrol-based plastics. Chitin-based plastics biodegrade quickly and easily, leaving little to no impact on the environment. Additionally, chitin is widely used for medical applications due to its biocompatibility and antimicrobial potential [12]. For applications in various industries, chitin is mainly converted to chitosan, a derivative, by deacetylation [13].

Chitosan, a biopolymer converted from chitin, is a material of economic interest due to its biodegradability, biocompatibility, non-toxicity, and bio-adhesiveness [10,14,15,16], which can be widely applied to medical fields [17,18], the food industry [19], agricultural [20], textiles [21], cosmetics [16], wastewater treatment plants [22,23], etc. Recently, the principal sources of raw material for the commercial-scale production of chitin and chitosan are crustacean exoskeletons, generally crabs and shrimps, of which there are around 100 billion tons worldwide [24,25]. However, as the demand for chitin and chitosan in various industries increases each year, many researchers have become interested in studying new sources of raw materials, such as insects and various shells, for reserved raw materials for production in the future [2,26,27]. Generally, the production of chitin and chitosan was performed via the chemical process [28] including four steps: demineralization, removing minerals using hydrochloric acid (HCl) to dissolve; deproteinization, removing proteins using sodium hydroxide (NaOH) to dissolve; decolorization, removing pigment using ethanol as a solvent; and deacetylation, removing acetyl groups with strong NaOH to convert to chitosan. In the case of mussel shell used as a raw material, the demineralization process will form Ca^2+^ and Cl^−^ ion aqueous solution. If this solution is not used, it becomes wastewater, but if it is used to prepare CaCl_2_ powder by an evaporation method, the obtained compound has a low price to sell and is used in minor industries [29]. For this reason, if acetic acid (CH_3_COOH) is used instead of hydrochloric acid (HCl) in the demineralization process, the resulting residual demineralization solution contains calcium and acetate ions, which can be used to produce a calcium acetate compound with the benefit of both high price and more use. Calcium acetate (Ca(CH_3_COO)_2_) is an interesting calcium compound due to its application in various industries, such as an absorbent of toxic gases in environmental fields [30], drugs for calcium deficiency and hyperphosphatemia patients in medical fields [31], stabilizers and preservatives in the food industry [32], plant micronutrient and soil adjuster in the agricultural industry [33], raw material for chemical production in the chemical industry [34,35,36], etc. Calcium acetate can be found in anhydrous, hemihydrate, and monohydrate forms, but the monohydrate is the most common form, which is its natural state [37]. Until now, calcium acetate has been produced by a reaction between acetic acid (CH_3_COOH) and various calcium sources, including calcium carbonate from lime rocks, eggshells, or mollusk shells (mussels, oysters, cockles, scallops, etc.) [37,38,39]. Production methods include precipitation [40], evaporation [41], replacement reaction [38], hydrothermal [42], and solvothermal processes [43]. Each production method’s differences in conditions (raw agents, pH, temperature, stirring, time, etc.) affect the product price as well as the chemical and physical properties [38,41]. However, previous works have lacked data on chitin/chitosan extraction and the simultaneous production of calcium acetate compounds from mussel shells.

This research reports on the chitin/chitosan extraction via the demineralization process by acetic acid, and the result was filtered to separate the demineralization solution and the solid. The demineralization solution, including Ca^2+^ and CH_3_COO^−^ ions, was evaporated to obtain dried calcium acetate powder for the first time. The solid was treated via three steps: deproteinization, decolorization, and deacetylation to obtain chitin and finally chitosan. The obtained chitin, chitosan, and calcium acetate samples were characterized to confirm their chemical and physical properties. This research supports a further step for developing a circular economic process of industrial-scale chitin, chitosan, and calcium acetate production that not only extracts value-added compounds from mussel shells but also promotes waste reduction and uses resources more efficiently.

## 2. Results and Discussion

### 2.1. Preparation Optimization Results

**Figure 1:**
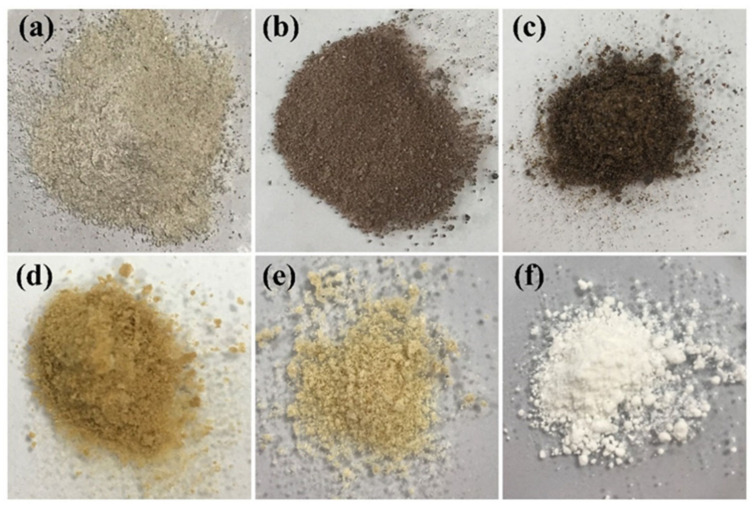
Photographs of mussel shell powders (**a**), RC (**b**), NC (**c**), PC (**d**), CS (**e**), and CA (**f**) extracted from mussel shells.

The colors of the mussel shells, raw chitin (RC), non-pure chitin (NC), pure chitin (PC), chitosan (CS), and calcium acetate (CA) powders shown in Figure 1 are white-brown, light black–gray, grayish black, dark brown, light brown, and white, respectively. The quantities of RC, NC, PC, and CS powders extracted from 100 g of mussel shell powder extracted were found to be 3.02, 2.98, and 2.70 g, respectively. The reduction in the amount is caused by the removal of impurities in each extraction process of RC to CS forms. The amount of chitin/chitosan obtained in this work was lower than that of chitin/chitosan from shrimp shells (7–14%) [44]. The degree of deacetylation (DD) of CS was 86%, which indicates a high degree of deacetylation. This value is close to those reported in previous studies, ranging from 83 to 93% by Triunfo et al. [27] and higher than the 32–52% reported by Kaewprachu and Jaisan [2]. A DD above 85% is generally considered suitable for applications requiring high solubility and cationic activity, such as biomedical materials, drug delivery systems, and antimicrobial films [20,27]. The molecular weight (Mw) of CS was estimated via intrinsic viscosity using the Mark–Houwink equation ($\left[\eta \right]=KM_{w}^{a}$) where K and α are constants that are characteristic for a particular polymer–solvent system at a specific temperature, and [η] is the intrinsic viscosity of the polymer in that solution, and it was calculated to be 72 kDa.

The weight of CA is 165.23 g, which was obtained from the evaporation of 1000 mL of the liquid fraction from the demineralization process. Based on the experiment data, the percentage yield of CA production was estimated according to Equation (1), which was found to be 96.06%.

CaCO_3_(s) + 2CH_3_COOH(aq) ⟶ Ca(CH_3_COO)_2_·H_2_O(s) + CO_2_(g) (1)

The mineral composition of the prepared calcium acetate was identified by X-ray fluorescence (XRF). The main composition found in calcium acetate monohydrate (Ca(CH_3_COO)_2_·H_2_O) is calcium oxide (CaO) with contents of 97.8% CaO. Other impurities found in calcium acetate monohydrate (Ca(CH_3_COO)_2_·H_2_O) include Na_2_O, MgO, Al_2_O_3_, SiO_2_, P_2_O_5_, SO_3_, Cl, K_2_O and SrO with contents of 1.2300%, 0.0822%, 0.0168%, 0.0218%, 0.0635%, 0.1480%, 0.0490%, 0.0121% and 0.6140%, respectively. The mineral composition of the prepared calcium acetate in this work shows a significantly higher CaO content (97.8%) compared to calcium acetate prepared from cockle shells (~95%) and oyster shells (~90%) in the previous research [38,39] due to the different production methods. Due to its high calcium content and the absence of toxic elements detected by XRF, the calcium acetate derived from the liquid fraction of the demineralization step in chitin extraction presents a sustainable and efficient alternative for calcium acetate production. This approach also helps minimize wastewater generation. Additionally, this research demonstrates the competitive potential of chitin and chitosan from green mussel shells as additional and alternative sources for the general commercial production obtained from crustaceans, affecting economic and environmental benefits to produce calcium compounds and the reduction in waste from chitin production processes.

### 2.2. The Fourier-Transform Infrared (FTIR) Results

**Figure 2:**
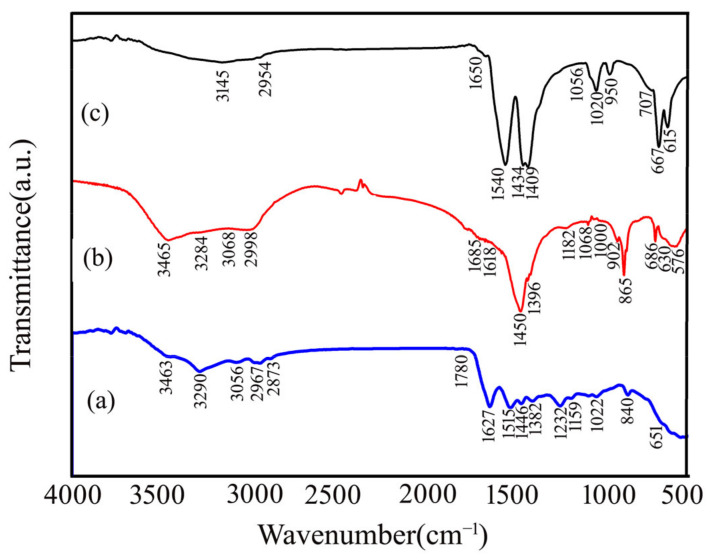
FTIR spectra of (**a**) pure chitin (PC), (**b**) chitosan (CS), and (**c**) calcium acetate. CA extracted from mussel shells.

The FTIR spectra of pure chitin (PC), chitosan (CS), and calcium acetate (CA) are present in Figure 2. The FTIR spectra of chitin (Figure 2a) and chitosan (Figure 2b) exhibit some characteristic bands: the typical band at 3463 cm^−1^ is attributed to -OH groups’ stretching vibration and intermolecular hydrogen bonding. The two weak bands, at 3290 and 3056 cm^−1^, are assigned to the asymmetric N-H (n_as_NH_2_) and symmetric N-H (n_s_NH_2_) stretching of amide groups in chitin, respectively. Weak bands at 2962 and 2873 cm^−1^ are assigned to asymmetry and symmetric C-H stretching (n_as_CH_3_), respectively. The characteristic bands of amide groups observed at 1627 and 1448 cm^−1^ for chitin (Figure 2a) are attributed to C=O stretching (amide I) and the bending mode CH_2_, respectively [45,46]. The characteristic bands of amide groups observed at 1508 and 1382 cm^−1^ for chitin (Figure 2a) are attributed to C-N stretching (C-N-H) + bending NH mode (amide II) and C-N stretching + bending NH mode (amide III), respectively [45,46]. For the FTIR spectrum of chitosan (Figure 2b), the vibrational bands of amide groups observed at 1627 and 1515 cm^−1^ corresponded to N-H bending (amide I) and C-N stretching (amide II), respectively. The difference in the number of bands and weak intensity bands in this region (1700–1200 cm^−1^) is caused by the elimination of the acetyl group (-COCH_3_), confirming the conversion of chitin to chitosan [47]. Bonds between 1200 and 1000 cm^−1^ are assigned to nC-C and vC-O modes, including a shoulder due to the n_as_ C-O-C of the glycosidic linkage (1226 cm^−1^), whilst the principal absorption is due to vC-O weakly coupled to δC-O-H. The intense band exhibited at 842 cm^−1^ is assigned as a C-H β-glycosidic bond [48,49]. Some peaks below 600 cm^−1^ are related to C-O (543/577 cm^−1^). The chitin and chitosan obtained from mussel shell extraction in this work are quite similar and correspond to those obtained from crustaceans and insects [44,50,51].

The FTIR spectrum of CA (Figure 2c) demonstrates the broad band at 3145 cm^−1^, which is assigned to the symmetric ν_s_ (O-H) and asymmetric ν_as_ (O-H) stretching modes of water (H_2_O) molecule overlap with vibrational stretching modes of the methyl (CH_3_) group of the acetate (CH_3_COO^−^) anion [52]. The weak peak at 1650 cm^−1^ contributes to the symmetric ν_2_(H-O-H) bending mode of H_2_O [37]. The strong peak at 1540 cm^−1^ is attributed to the asymmetric ν_as_ (C-O) stretching, whereas two peaks at 1434 and 1409 cm^−1^ represent the symmetric ν_s_ (C-O) stretching of acetate anion [53]. For the weak peak at 1020 cm^−1^, the out-of-plane δ_op_ (CH_3_) stretching and in-plane δ_ip_ (CH_3_) bending vibrational mode of the CH_3_ group was observed. Meanwhile, the ν (C-C) stretching vibration of the C-C bond was exhibited at 950 cm^−1^. A medium peak at 667 cm^−1^ is assigned to the symmetric twisting δ_st_ (O–C-O) and rocking δ_sr_ (O-C-O) vibration of O-C-O, whereas a medium peak at 615 cm^−1^ is assigned as the out-of-plane δ_op_ (O-C-O) stretching vibration of O-C-O. This result is consistent with the FTIR spectra of calcium acetate prepared by other raw materials such as eggshells, cockle shells, and oyster shells in previous works [38,39,54].

### 2.3. X-Ray Diffraction (XRD) Results

**Figure 3:**
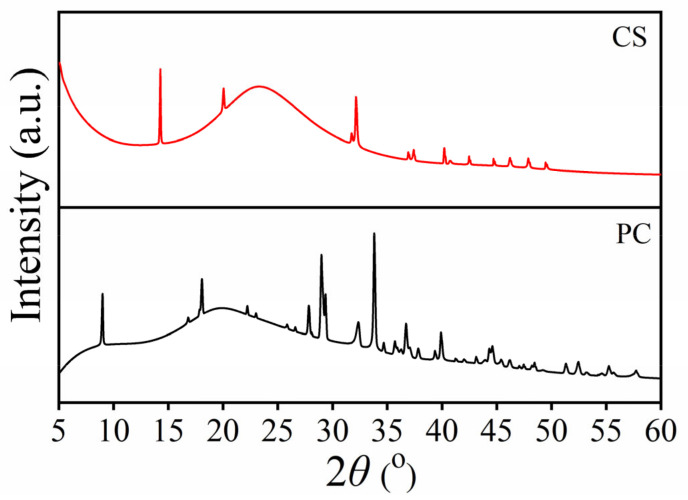
XRD patterns of pure chitin (PC) and chitosan (CS) extracted from mussel shells.

The XRD patterns of pure chitin (PC) and chitosan (CS) are shown in Figure 3. The XRD patterns of the obtained PC and CS samples are similar to the chitin and chitosan extracted from crustacean shells and insects in previous research [44,50,51]. According to the literature [47,48], chitin has classic sharp peaks at 9, 19, 27, 28, 29 and 34^o^, which correspond to reflections (020), (021), (013), (120), (130), and (110) of α-chitin crystals, respectively. The comparison of our XRD result with different types of chitin crystals showed that it is similar to and ascribed to chitin (JCPDS card no: 39-1894) [44,50,51,55]. The XRD pattern of chitosan (CS) is given in Figure 3. The three sharp peaks were observed at 14, 20, and 33°, which correspond to reflections of (020), (110), and (130), respectively [47,50]. This result is a good match with the chitosan XRD patterns (chitosan isolated from organisms such as shrimp, crab, and insects) reported by Yen et al. [56] and Suneeta et al. [57] However, obvious changes were observed in the reflection angle position between chitin and chitosan prepared by mussel shell in this work.

**Figure 4:**
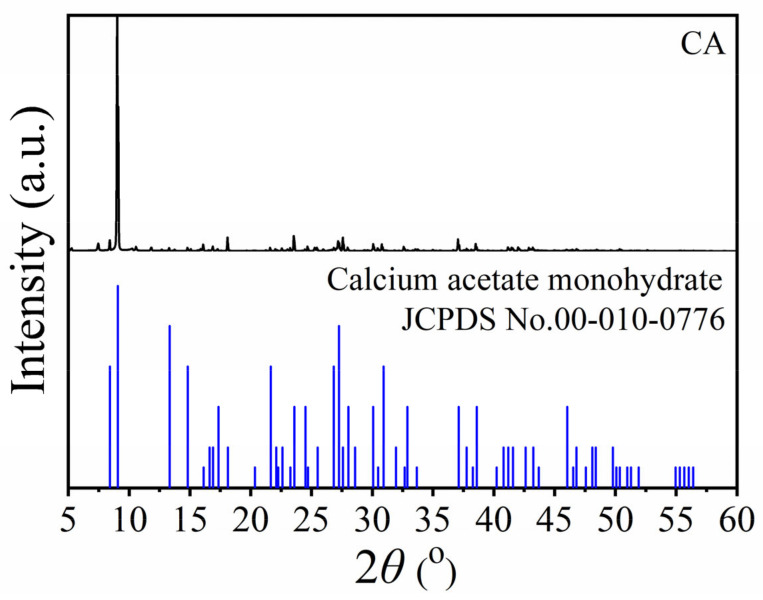
XRD pattern of calcium acetate (CA) from mussel shells.

Figure 4 shows the X-ray diffraction (XRD) pattern of the calcium acetate (CA). The XRD pattern of CA clearly shows that the crystal structure of the obtained product corresponded to the calcium acetate monohydrate (Ca(CH_3_COO)_2_·H_2_O) of the JCPDS data (PDF # 010–0776) [52]. The XRD pattern of the sample is similar to calcium acetate prepared from eggshells, cockle shells, and oyster shells in previous works [38,39,54]. The crystalline sizes of CA based on the XRD patterns of Ca(CH_3_COO)_2_⋅H_2_O calculated by Scherrer’s equation (S_c_ = 0.9λ/β·cosθ), where λ is the employed X-ray wavelength (0.154059 nm), β is the full width at the half maximum (FWHM in radians) of the investigated diffraction peak, and the result was found to be 72.30 nm.

### 2.4. Thermogravimetric Analysis (TGA) Results

**Figure 5:**
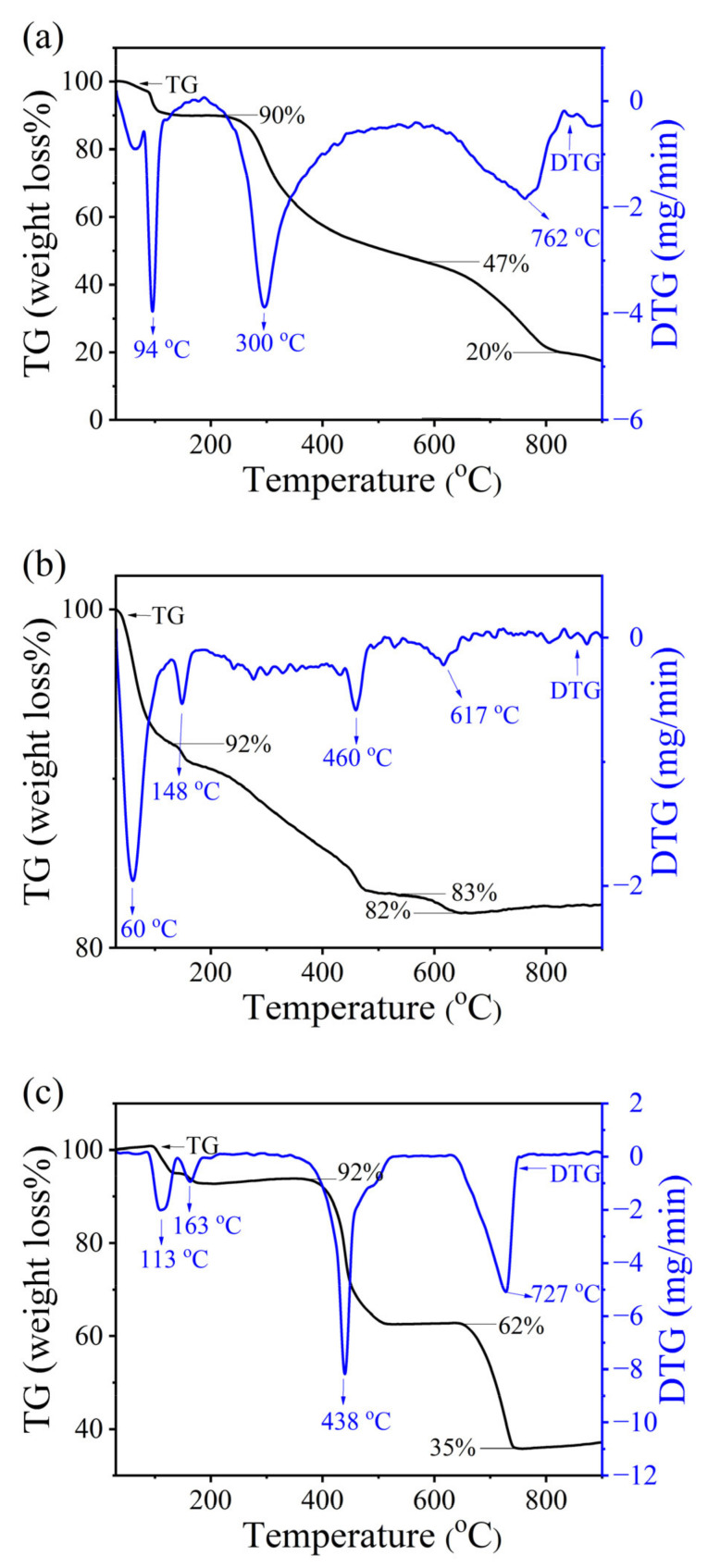
TG/DTG curves of (**a**) pure chitin (PC), (**b**) chitosan (CS), and (**c**) calcium acetate (CA) extracted from mussel shells.

**Figure 6:**
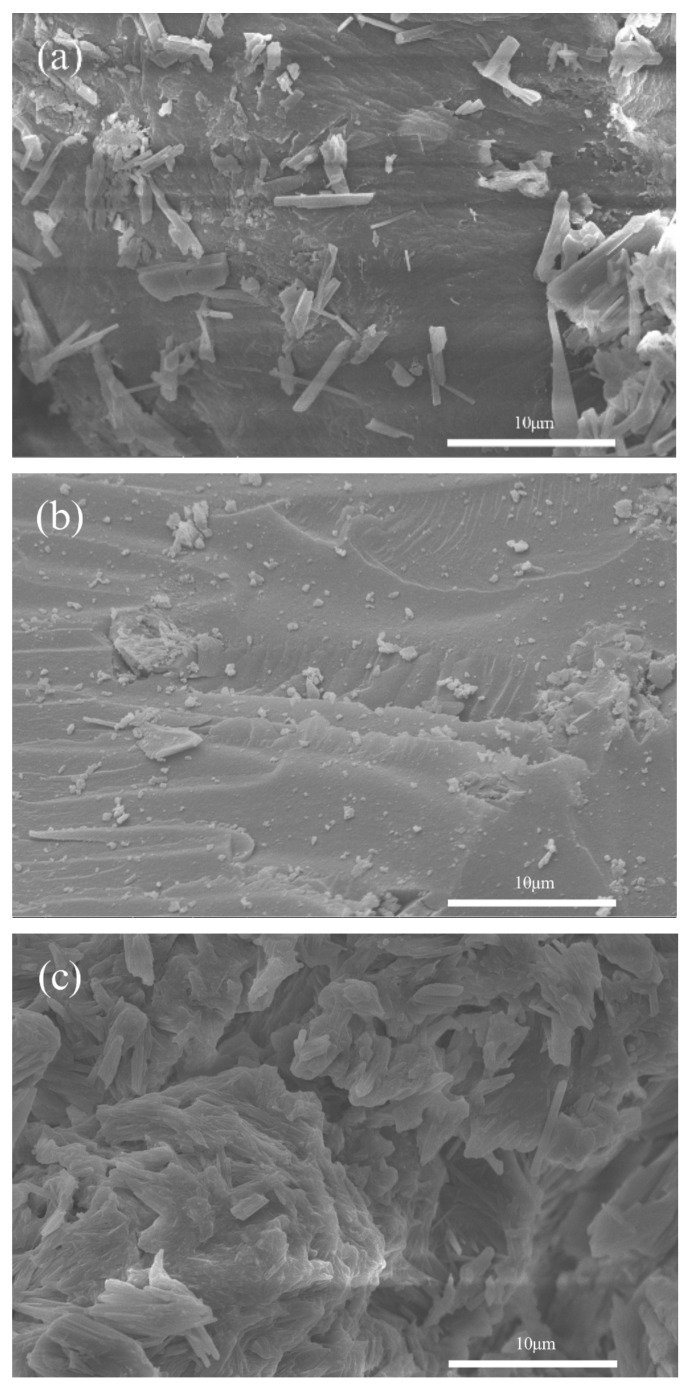
SEM images of (**a**) pure chitin (PC), (**b**) chitosan (CS), and (**c**) calcium acetate (CA) extracted from mussel shells.

The TG and DTG curves of pure chitin (PC) and chitosan (CS) extracted from mussel shells are shown in Figure 5. The TGA results of the PC (Figure 6a) and CS (Figure 6b) are very similar, which depict their thermal stabilities. The TG curves of the PC (Figure 5a) and CS (Figure 5b) were classified into 3 regions as 50–200, 200–480, and 480–820 °C, which relate with the three DTG peaks at 150, 300, and 780 °C, respectively. The first decomposition region, found to be a weight loss of 6% for PC and 10% for CS, could be attributed to the evaporation of water molecules associated with the hydrophilic groups in the samples [58,59]. The weight loss of the PC and CS observed in the second decomposition region was found to be the same value (44%), which is attributed to the degradation of the saccharide structure of the molecule, including the breakdown of acetylated glucosamine units and the breakdown of the polymer chains. For the third decomposition region, the weight losses of 25% for PC and 23% for CS were due to the carbonization of residual organic matter, which subsequently results in the formation of volatile compounds including acetic acid (CH_3_COOH), butyric acid (CH_3_CH_2_CH_2_COOH) and a series of lower fatty acids where C2, C3 and C6 predominate [60,61,62,63]. The remaining residue, about 18% for PC and 25% for CS above 800 °C, was mostly due to the formation of an inorganic complex containing C, N, and O [62,64]. The TGA results of chitin and chitosan obtained from mussel shells in this work are like those of products prepared from crustaceans in previous works [58], indicating the thermal stabilities of the prepared samples.

The TG/DTG curves of calcium acetate (CA) are shown in Figure 5c. The TG curves of CA were classified into 3 regions as 50–200, 350–520, and 650–780 °C. The first decomposition step was found to be a weight loss of 8% corresponding to the evaporation of water (dehydration process) from Ca(CH_3_COO)_2_⋅H_2_O, and the thermal decomposition product was formed as anhydrous Ca(CH_3_COO)_2_, as shown in Equation (2) [37]. The corresponding DTG curves are shown as double peaks at 113 and 163 °C, which is the effect of the inter- and intramolecular interaction of water molecules with the different surroundings in this CA structure [37]. As the second thermal decomposition process (DTG peak at 438 °C), the weight loss of about 30% is assigned to the decomposition of anhydrous Ca(CH_3_COO)_2_, which then thermally generated the remaining CaCO_3_ solid and the evolved acetone (CH_3_COCH_3_) gas [37]. At the same time, the generated CH_3_COCH_3_ further decomposed immediately to ketene (C_2_H_2_O) and methane (CH_4_). The second decomposition step (Equation (3)) was called the “deacetonation process” [37]. The third thermal decomposition step (DTG peak at 727 °C), correlating to the weight loss of about 27%, is attributed to the thermal decomposition of CaCO_3_, which then generates the remaining solid-state CaO and the evolved CO_2_ gas. The third decomposition step (Equation (4)) was called the “decarbonization process” [37].

Ca(CH_3_COO)_2_·H_2_O(s) ⟶ Ca(CH_3_COO)_2_(s) + H_2_O(g) (2)

Ca(CH_3_COO)_2_(s) ⟶ CaCO_3_(s) + CH_3_COCH_3_(g) (3)

CaCO_3_(s) ⟶ CaO(s) + CO_2_(g) (4)

The total mass loss of 67% relates to the mass retention of 33% (>720 °C), forming CaO as a final stable compound, which was consistent with theoretical data as 68% and 32% according to reactions, respectively. However, this result may not agree with Ca(CH_3_COO)_2_⋅H_2_O obtained from other calcium raw materials (oyster shells (>800 °C) [65] and scallop shells (>700 °C) [66] with another preparation method reporting a higher temperature to obtain the CaO.

### 2.5. Scanning Electron Microscope (SEM) Results

The surface morphologies of pure chitin (PC), chitosan (CS), and calcium acetate (CA) extracted from mussel shells were investigated with SEM analysis; the results are shown in Figure 6. The morphology of PC (Figure 6a) shows smooth surfaces with timber-like microparticles on the surfaces. In contrast, the morphology of CS (Figure 6b) shows a smooth surface, which is similar to the results of commercial chitosan from shrimp shells in previous studies [67] without timber-like particles. It can be indicated that the strong base used in the deacetylation process not only converted chitin to chitosan by removing the acetyl group (−COCH_3_) but also removed some particles on the surface of the prepared biopolymer.

The SEM image of calcium acetate (CA) is shown in Figure 6c. The morphology result of CA shows the coalescence of swiftlet nest-like particles. The morphology (size and shape) of the synthesized Ca(CH_3_COO)_2_⋅H_2_O in this work is significantly different from those produced from another raw agent with another synthesis method in our previous report [38,39], which exhibited rod-like particles with significantly larger sizes.

## 3. Materials and Methods

### 3.1. Materials and Reagents

Mussel shells were collected from a seafood market in Chonburi, Thailand. The collected mussel shells were rinsed thoroughly with distilled water and dried in an oven at 100 °C for 2 h. After thorough cleaning and drying, the mussel shells were ground into a fine powder and sieved through a 100 mesh sieve to obtain mussel shell powders.

Commercial-grade chemicals, 99.85% *w*/*w* (17.42 M) acetic acid (CH_3_COOH) and 98% sodium hydroxide pellets (NaOH), and 50% *w*/*v* hydrogen peroxide, were diluted for use in the experiment without further purification. Acetic acid (CH_3_COOH) was diluted to 1 M with deionized water. Sodium hydroxide pellets (NaOH) were dissolved in deionized water to obtain a 10 M sodium hydroxide solution used in the deacetylation and then diluted to 1 M to aid in the deproteinization. Finally, hydrogen peroxide was diluted with deionized water to 10% *w*/*v* to apply for the decolorization.

### 3.2. Chitin Extraction by Demineralization

A demineralization process was performed to eliminate the mineral components in mussel shells using acetic acid to incur a demineralization solution containing calcium (Ca^2+^) and acetate (CH_3_COO^−^) ions to produce calcium acetate in the next step. Typical procedure: First, 100 g of mussel shell powders was mixed with 1000 mL of 1 M acetic acid with the magnetic stirrer at a speed of 600 rpm for 2 h. The suspension was filtered to separate solid and liquid fractions, which are used in the next steps. The solid fraction was washed to neutral pH with deionized water and then dried in an oven at 60 °C for 2 h to obtain raw chitin (RC). The liquid fraction was then used to prepare the calcium acetate powders.

### 3.3. Chitin Purification

− Deproteinization

Eliminating the protein way, 1 g of RC was added into 10 mL of 1 M sodium hydroxide with a stirring speed of 600 rpm for 1 h. Then, the mixture was filtered to obtain powder, which was washed to neutral pH with deionized water and dried in an oven at 60 °C for 2 h. The powder obtained is non-pure chitin (NC).

− Decolorization

Removing pigments route, 1 g of NC was mixed with 10 mL of 10% w/v hydrogen peroxide (H_2_O_2_) under a stirring speed of 600 rpm and temperature at 90 °C for 30 min. Then, the result was filtered to obtain powder, washed to neutral with deionized water, and finally dried in an oven at 60 °C for 2 h. The brown powder obtained is pure chitin (PC).

### 3.4. Deacetylation

Converting chitin to chitosan by a deacetylation process, 1 g of PC was added into 20 mL of 10 M sodium hydroxide under a stirring speed of 600 rpm and temperature at 100 °C for 4 h. The suspension obtained was mixed with 99% ethyl alcohol in the ratio of 1:1 (*v*/*v*) in a continuous stirrer for 30 min. A light brown powder was obtained and isolated by filtration, washed with acetone three times, and dried at 60 °C for 2 h. The obtained light brown powder is chitosan (CS).

### 3.5. Calcium Acetate Production

The 1000 mL of the liquid fraction obtained from the demineralization process contains calcium (Ca^2+^) and acetate (CH_3_COO^−^) ions. The calcium content was analyzed by EDTA titration and calculated to be 0.96 M, revealing a limiting reagent. This solution was evaporated in an oven at 60 °C for 24 h to obtain dried white powder and then ground and sieved through 100 mesh to obtain fine white powders of calcium acetate labeled as CA.

### 3.6. Characterization

The prepared pure chitin (PC), chitosan (CS), and calcium acetate (CA) were analyzed to confirm their quality and suitability for further applications. A Fourier transform infrared (FTIR) spectrophotometer (Spectrum GX, PerkinElmer Inc., Waltham, MA, USA) was used to record the IR spectra of samples from 4000 to 400 cm^−1^ using a resolution of 4 cm^−1^. For sample preparation, about 1 g of the sample was mixed homogeneously with 10 g of spectroscopic-grade potassium bromide (KBr) and pressed to form small pallets for FTIR measurement [68]. An X-ray diffractometer (XRD) instrument (MiniFlex, Rigaku Corporation, Tokyo, Japan) was used to investigate the crystal structure of the samples with Cu Kα irradiation (40 kV, 32 mA) from a scan angle between 5° and 60° at a scan speed of 0.04° s^−1^. A thermogravimetric analyzer (Pyris Diamond, PerkinElmer Inc., Waltham, MA, USA) was employed to investigate the thermal behavior of the samples. About 30 mg of each sample was placed into α-Al_2_O_3_ pans under nitrogen (N_2_) atmosphere with a flow rate of 100 mL⋅min^−1^ between 30 and 900 °C. Using a sputtering technique, a scanning electron microscope (LEO 1530, Carl Zeiss AG, Oberkochen, Germany) was used to examine the morphologies of samples after coating with gold powder.

## 4. Conclusions

This research successfully utilized mussel shell waste as a renewable source of biopolymer as chitin/chitosan and advanced calcium compound as calcium acetate. The chemical and physical properties of the prepared chitin, chitosan, and calcium acetate were characterized by FTIR, XRD, TGA, and SEM techniques. The properties of all of the obtained products are similar to chitin and chitosan prepared from crustaceans and insects in previous research, but the yield is lower. In contrast, the prepared calcium acetate has similar characteristics (the vibrational modes, crystal structure, and thermal behavior) to this compound prepared from other calcium sources by another method of preparation reported in the literature, but the purity of the product in this work is higher (97.8% CaO) without non-toxic elements. These results show the potential of the sustainable production process of chitin, chitosan, and calcium acetate from mussel shells as alternative natural polymers and calcium sources for industrial applications. Moreover, the developed process leads to a circular economy process, which can reduce wastewater in the production process of chitin and chitosan, reduce environmental problems from food waste, produce more valuable products, and use resources more efficiently.

## Data Availability

All data are fully available without restriction.
